# Supplementary material for: Metabolic syndrome and idiopathic sudden sensori-neural hearing loss
Source: PLoS One. 2020 Aug 28;15(8):e0238351. doi: 10.1371/journal.pone.0238351 (PMC7454951; doi:10.1371/journal.pone.0238351)
Supplement: Attachment — Submitted filename: response to reviewers.doc [file pone.0238351.s002.doc]

Rinaldi et al. PONE-D-20-16260

**“Metabolic Syndrome and Idiopathic Sudden Sensori-Neural Hearing Loss”**

*We thank the Editor and the Reviewer for their positive and constructive comments and based on their suggestions, the manuscript has been revised. The comments were very well taken and the consequent changes definitively ameliorated the quality of the manuscript. Here we are submitting a revised version of our manuscript based on their suggestions. To address the Reviewer’ concerns we have provided new information that fully supports our model and any predictions that it makes. Our detailed response to each of the Reviewers’ points is reported below.*

**Journal Requirements**

1. Please ensure that your manuscript meets PLOS ONE's style requirements, including those for file naming. The PLOS ONE style templates can be found at<https://journals.plos.org/plosone/s/file?id=wjVg/PLOSOne_formatting_sample_main_body.pdf> and <https://journals.plos.org/plosone/s/file?id=ba62/PLOSOne_formatting_sample_title_authors_affiliations.pdf>

*The PLOS ONE style requirements have been followed*

2. In the ethics statement in the manuscript and in the online submission form, please provide additional information about the patient records used in your retrospective study, including:  a) the date range (month and year) during which patients' medical records were accessed; and b) the source of the medical records analyzed in this work (e.g. hospital, institution or medical center name).

*Additional information have been provided in the Ethics Statement*

3. We noticed minor instances of text overlap with the following previous publication(s), which need to be addressed:

(1) <https://worldwidescience.org/topicpages/m/metabolic+syndrome+involvement.html>

(2) <https://en.wikipedia.org/wiki/Metabolic_syndrome>
 The text that needs to be addressed involves the (1) Introduction (lines 53-55), (2) Introduction section (lines 58-64).

*In the Introduction Lines 53-55 and 58-64 have been rephrased even if they are the Diagnostic criteria of the disease and cannot be dramatically changed.*

4. We note that you have indicated that data from this study are available upon request. PLOS only allows data to be available upon request if there are legal or ethical restrictions on sharing data publicly. For information on unacceptable data access restrictions, please see [http://journals.plos.org/plosone/s/data-availability#loc-unacceptable-data-access-restrictions](http://journals.plos.org/plosone/s/data-availability#loc-unacceptable-data-access-restrictions).

In your revised cover letter, please address the following prompts:

a) If there are ethical or legal restrictions on sharing a de-identified data set, please explain them in detail (e.g., data contain potentially identifying or sensitive patient information) and who has imposed them (e.g., an ethics committee). Please also provide contact information for a data access committee, ethics committee, or other institutional body to which data requests may be sent.

b) If there are no restrictions, please upload the minimal anonymized data set necessary to replicate your study findings as either Supporting Information files or to a stable, public repository and provide us with the relevant URLs, DOIs, or accession numbers. Please see <http://www.bmj.com/content/340/bmj.c181.long> for guidelines on how to de-identify and prepare clinical data for publication. For a list of acceptable repositories, please see [http://journals.plos.org/plosone/s/data-availability#loc-recommended-repositories](http://journals.plos.org/plosone/s/data-availability#loc-recommended-repositories).

We will update your Data Availability statement on your behalf to reflect the information you provide.

*Data set has been uploaded*

In relation the changes requested by the Editor the following changes have been made.

NOVELTY: There are other similar studies in the literature, with larger number of patients. Bottomline - what does your study add to the already existing literature?

*In the discussion a paragraph that highlights the fact that this is the first study that correlates abdominal obesity with hearing recovery has been added*

RELEVANCE: What literature gap does this study aim to fill? This matter needs to be explored in-depth in the introduction of the manuscript.

*In the Introduction the pathophysiological hypothesis of ISSHL have been added. In addition the following phrase has been added “Chien et al [12] in a case control study have reported that MetS increase the risk of sudden hearing loss and that the risk increase with the number of MetS components. In addition the presence of MetS has been associated with poorer hearing recovery by other authors [14-16] that however have not used central obesity as a criterion for MetS. “*

FOCUS: Although the authors defined a study design aimed to evaluate the correlations between cardiovascular diseases and ISSNHL, there is an extensive discussion on findings from other authors and speculation on underlying pathophysiological mechanisms. Although important and informative, I recommend including a more in-depth analysis of the authors' own findings.

*In the discussion author’s own findings have been discussed*

DATA AVAILABILITY: I strongly recommend the authors to make their raw data available as a supplementary material.

**Reviewer #1:**

I read the manuscript entitled “Metabolic Syndrome and Idiopathic Sudden Sensorineural Hearing Loss” with great interest.
The authors aimed to evaluate the impact of Metabolic Syndrome (MetS) and Idiopathic Sudden Sensorineural Hearing Loss (ISSHL) and the association between the presence of MetS and ISSHL.
Major issues:
Introduction
1. The pathophysiology of ISSHL remains unknown and we have a lot of widely accepted theories. In cases of ISSHL resulting from a known intravascular insult, the loss is permanent. However, the authors describe a high rate of spontaneous recovery suggesting a vascular mechanism at the basis of this disease.

*In the Introduction the following paragraph has been added “Viral infections, vascular occlusions, immune-mediated mechanisms and cochlear membrane breaks have been proposed as pathogenic mechanisms, however clinical and experimental evidence that confirm one or other mechanisms is lacking [Merchant SN, Durand ML, Adams JC. Sudden deafness: is it viral?. ORL J Otorhinolaryngol Relat Spec. 2008;70(1):52-62. doi:10.1159/000111048]. And the phrase referring to the spontaneous recovery has been erased.*

2. The lack of studies on the association between ISSHL and the subsequent risk for stroke prevents from simultaneous contributions of vascular, biochemical and metabolic factors.

*In Introduction the phrase “suggesting that hearing loss may share similar etio-pathogenic mechanisms.” Has been added*

Methods
1. Subjects: 39 patients with ISSHL and 44 control– small size sample.

*We acknowledge that the sample is not very large*

2. What comorbidities did the patients have previously? Have these variables been taken into account?

*All patients affected by by acoustic neuroma, central lesions, autoimmune disorders, Meniere’s disease, multiple sclerosis, trauma, medication, noise, or prior ear surgery were excluded. While comorbidities are reported in Table 1*.

3. The authors describe that all patients underwent the same treatment protocol (steroids, carbogen inhalation, pentoxifylline, vitamin C and magnesium sulfate. Is that treatment regimen a clinical protocol for treating patients with ISSHL in your hospital?

*Yes this is the standard protocol at our hospital*

4. It is know that standard pure tone audiometry not only provides the criteria for diagnosis of ISSHL, characteristics of the initial audiogram have prognostic value. Have these variables been taken into account?

*The shape of the audiogram has not bee evaluated due to the small sample size*

Results
1. There are several studies in the literature who presented similar results, with larges casuistic. For example: Jung SY, Shim HS, Hah YM, Kim SH, Yeo SG. Association of Metabolic Syndrome With Sudden Sensorineural Hearing Loss. JAMA Otolaryngol Head Neck Surg. 2018;144(4):308–314. doi:10.1001/jamaoto.2017.3144.

*Jung et al has already been cited,*

Discussion
1. Most of the discussion only describe findings from other authors.

*In the discussion the following paragraph has been added that refers to previous studies on the same topic*

Reviewer #2:

This manuscript entitled: "Metabolic Syndrome and Idiopathic Sudden Sensorineural Hearing Loss" is well written and addresses a topic that is being widely discussed in the literature. however, we highlight some details that need to be clarified:
1) in the introduction, the authors approach that the pathogenesis of idiopathic sudden hearing loss is controversial, however suggest only a vascular mechanism at the basis of this disease and discussed that ISSHL endothelial dysfunction has been reported "basing all the reasoning for only vascular causes and I think it is important to emphasize that the pathogenesis of ISSHL has multiple other physopathogenic processes involved.

*In the Introduction the following paragraph has been added “Viral infections, vascular occlusions, immune-mediated mechanisms and cochlear membrane breaks have been proposed as pathogenic mechanisms, however clinical and experimental evidence that confirm one or other mechanisms is lacking [Merchant SN, Durand ML, Adams JC. Sudden deafness: is it viral?. ORL J Otorhinolaryngol Relat Spec. 2008;70(1):52-62. doi:10.1159/000111048]. And the phrase referring to the spontaneous recovery has been erased.*

2) The objective is very clear "we evaluated the association between the presence of MetS and ISSHL,." and the study method is all based on a study of association and not a cause and effect relationship. For this reason the statistical tests performed were all for association between two injuries, (sudden idiopathic neurosensory loss and the metabolic syndrome) whose sample is all taken in an association study. The following conclusion is not supported by the results:
"The presence of MetS increased the risk of ISSHL" or "Endothelial dysfunction and microvascular damage could be related to both increased prevalence and poorer recovery". The methodology used in this study as well as the sample size and statistical analysis does not allow us to make these conclusions. We can only conclude that there is an association between ISSHL and metabolic syndrome and that this association is negatively influenced hearing recovery in ISSHL patients.
*The Abstract has been edited as follows “MetS was associated with ISSHL and this association negatively influenced the hearing recovery of these patients”*

*The phrase “Endothelial dysfunction and microvascular damage could be related to both increased prevalence and poorer recovery.” has been removed*

3) In the abstract the acronym meaning BMI and WHR needs to be scoredin the abstract the acronym meaning BMI and WHR needs to be scored.

*The acronym has been scored*
